# Editorial: Chemical Innovative Approaches in Cancer Molecular Medicine and Translational Clinical Research

**DOI:** 10.3389/fchem.2020.00820

**Published:** 2020-09-03

**Authors:** Rosaria Benedetti, Mariarosaria Conte, Carmela Dell'Aversana, Finn K. Hansen, Clemens Zwergel

**Affiliations:** ^1^Department of Precision Medicine, University of Campania Luigi Vanvitelli, Naples, Italy; ^2^Institute Experimental Endocrinology and Oncology “Gaetano Salvatore”, National Research Council (CNR), Naples, Italy; ^3^Department of Pharmaceutical and Cell Biological Chemistry, Pharmaceutical Institute, University of Bonn, Bonn, Germany; ^4^Department of Drug Chemistry & Technologies, Sapienza University of Rome, Rome, Italy

**Keywords:** cancer, epigenetics, drug combination, multitarget compounds, target therapy

Cancer is considered a multifactorial pathology, whose understanding involves genomic and epigenomic studies supplemented by biochemical, biological, molecular, and epidemiological data. Current cancer research strategies are based on the paradigm of “targeted” therapies. Targeted cancer therapies are drugs or other substances that block the growth and spread of cancer by interfering with specific molecules (“molecular targets”) that are involved in growth, progression, and spread of cancer. Many targeted cancer therapies have been approved by the Food and Drug Administration (FDA) to treat specific types of cancer. Others are being studied in advanced clinical trials, and many more are in preclinical and clinical testing. Despite recent progress in regression or control for a wide variety of tumors, some cancers do not respond to current therapeutic patterns, showing limited 5-year survival rates, high recurrence, and frequent relapse and metastases, making them big killers. Indeed, combined efforts between biologists, chemists, and oncologists are required to provide novel therapeutic options for patients and to achieve precision medicine based on the molecular integrated metabolic and (epi)genome signature.

A big deal of results has been published regarding cancers and their treatments, thus representing a very prosperous area of drug discovery. Despite the significant amount of drug discoveries in the vast field of cancer therapy, there is still an urgent need for novel and innovative treatments. Efficacy and safety of the therapy are a major concern and significant advances in structural biology and bioinformatics in the last 20 years gave medicinal chemists a well-filled toolbox for the design of innovative drugs using the most advanced techniques working closely together with biochemists, biologists, and other medical-related researchers to develop next generation anticancer therapies. In the present special issue, various aspects of modern approaches in cancer therapy have been either summarized in comprehensive review articles or published as original articles.

Regarding receptor-based ligands which gained more and more interest recently, Listro et al. explored the chemical space around their previously identified RC-106, a σ-receptor modulator. They found three novel potential anticancer agents via a combinatorial approach endowed with potent activity in the somewhat rare cancers such as glioblastoma and multiple myeloma (Listro et al.).

Souto et al. designed and synthesized purpurogallin-inspired inhibitors acting on the JumonjiC, a histone lysine demethylase (KDM4A), an innovative epigenetic target. The most potent analog displayed a considerable KDM4A inhibitory activity in *in vitro* models of colon cancer cells and the most potent antitumor action in several solid and hematological human cancer cell lines with no toxic effect in healthy cells (Souto et al.).

It is well-known that EGFR and VEGFR-2 represent promising targets for cancer treatment as they play a crucial role in tumor growth, angiogenesis, and metastasis. Saleh et al. developed novel fused pyrazole derivatives as EGFR and VEGFR-2 dual inhibitors active in liver cancer with IC_50_ values in the very low micromolar range for both enzymes. This paper is a valuable example of an innovative multitarget approach.

Di Bello et al. analyzed the recent advances in the use of statins, traditionally applied in cardiovascular diseases, to reduce lipid levels, applied in cancers. Indeed, several studies have demonstrated that these old drugs simvastatin, fluvastatin, and lovastatin are implicated in different pathways that enhance the survival time of patients with cancer under treatment in combination with antineoplastic agents (Di Bello et al.).

Transcription and translation are fundamental cellular processes that govern the protein production of cells. Laham-Karam et al. shed light on the recent advances regarding these processes upregulated in cancer and potential drug targets such as the transcription factors and RNA molecules. They took not only in consideration natural and synthetic compounds, but also DNA and RNA based approaches (Laham-Karam et al.).

The RNA-guided clustered regularly interspaced palindromic repeats (CRISPR)/associated nuclease 9 (Cas9)-based genome editing technology has increasingly become a recognized method for translational research. Pomella and Rota summarized in their mini-review an overview of the major aspects of the technology mentioned above with a focus on a group of rare pediatric malignancies, soft tissue sarcomas, on which this approach is having promising results.

Pane et al. provided insights on the recent innovative findings in non-muscle-invasive bladder cancers. They discuss novel immunotherapeutic options, new promising bladder-preserving treatments, as well as ongoing clinical trials (Pane et al.).

Besides interesting medicinal chemistry findings and innovative approaches in chemistry and biochemistry, other valuable approaches, such as targeted therapies in combination with one or more traditional chemotherapeutic agents, with the goal to reduce the toxicity and increase the efficacy, were either described in original articles or well-summarized in the concise reviews. To sum up, this special issue established ties between chemistry and oncology, providing some valuable and innovative approaches in molecular medicine and translational preclinical and clinical research.

## Author Contributions

All authors listed have made a substantial, direct and intellectual contribution to the work, and approved it for publication. All authors conceived and designed the editorial contribution and equally participated to the writing and editing.

## Conflict of Interest

The authors declare that the research was conducted in the absence of any commercial or financial relationships that could be construed as a potential conflict of interest.

